# Social Defeat Stress during Early Adolescence Confers Resilience against a Single Episode of Prolonged Stress in Adult Rats

**DOI:** 10.3390/cells10020360

**Published:** 2021-02-09

**Authors:** Giulia Federica Mancini, Enrico Marchetta, Irene Pignani, Viviana Trezza, Patrizia Campolongo

**Affiliations:** 1 Department of Physiology and Pharmacology, Sapienza University of Rome, 00185 Rome, Italy; giuliafederica.mancini@uniroma1.it (G.F.M.); enrico.marchetta@uniroma1.it (E.M.); pignani.1677271@studenti.uniroma1.it (I.P.); 2 Neurobiology of Behavior Laboratory, Santa Lucia Foundation, 00143 Rome, Italy; 3 Department of Science, Section of Biomedical Sciences and Technologies, University Roma Tre, 00146 Rome, Italy; viviana.trezza@uniroma3.it

**Keywords:** early-life stress, two-hit model, psychopathologies, BDNF, glucocorticoids

## Abstract

Early-life adverse experiences (first hit) lead to coping strategies that may confer resilience or vulnerability to later experienced stressful events (second hit) and the subsequent development of stress-related psychopathologies. Here, we investigated whether exposure to two stressors at different stages in life has long-term effects on emotional and cognitive capabilities, and whether the interaction between the two stressors influences stress resilience. Male rats were subjected to social defeat stress (SDS, first hit) in adolescence and to a single episode of prolonged stress (SPS, second hit) in adulthood. Behavioral outcomes, hippocampal expression of brain-derived neurotrophic factor, and plasma corticosterone levels were tested in adulthood. Rats exposed to both stressors exhibited resilience against the development of stress-induced alterations in emotional behaviors and spatial memory, but vulnerability to cued fear memory dysfunction. Rats subjected to both stressors demonstrated resilience against the SDS-induced alterations in hippocampal brain-derived neurotrophic factor expression and plasma corticosterone levels. SPS alone altered locomotion and spatial memory retention; these effects were absent in SDS-exposed rats later exposed to SPS. Our findings reveal that exposure to social stress during early adolescence influences the ability to cope with a second challenge experienced later in life.

## 1. Introduction

Stress exposure can lead to the development of several psychiatric diseases, including anxiety, depression, and post-traumatic stress disorder (PTSD) [1,2]. Early-life stressful experiences may lead to coping strategies that match or mismatch later-life adverse experiences, resulting in resilience or vulnerability, respectively, to the development of psychopathologies in adulthood [3,4,5,6]. Because adolescence is a crucial developmental stage associated with profound changes in the structure and function of the brain [7,8,9], stress experienced during this critical developmental period has more detrimental effects compared with those experienced in adulthood. The hypothalamic–pituitary–adrenal axis is still immature in early adolescence, and the release of cortisol (corticosterone in rodents) in response to acute stressors is higher than that in adulthood [10,11,12]. Chronic glucocorticoid exposure during adolescence is associated with adverse consequences, particularly in the hippocampus where it causes neuronal cell damage and dendritic atrophy, reduces neurogenesis, impairs synaptic plasticity, and suppresses long-term potentiation [13,14,15,16]. Brain-derived neurotrophic factor (BDNF), a member of the neurotrophin family, regulates axonal guidance, enhances synaptic plasticity and neurite outgrowth, and facilitates long-term potentiation [17]. Glucocorticoids strictly regulate the expression and signaling of BDNF, and a growing body of evidence indicates that a stress-induced change in BDNF signaling is a potential cause of stress-related disease [18,19,20,21]. Patients with depression or bipolar disorder have altered hippocampal BDNF expression [22], and patients with depression or PTSD have reduced hippocampal volume [23,24]. 

Adolescence is characterized by increased social interactions, as well as play and novelty-seeking behaviors, which are necessary for acquiring the skills related to developing autonomy and independence from parental caretakers [8]. Bullying and subordination episodes are social stressors that frequently occur among adolescents [25,26]. Numerous studies report that adolescent victims of bullying are more predisposed to mental disorders later in life, particularly anxiety and depression [27]. Human data reporting the influence of experiencing bullying during adolescence on the development of psychiatric disorders and their seriousness after additional trauma in adulthood are lacking. Understanding how early-life social stress could change an individual’s responsiveness to additional trauma later in life will facilitate the development of treatments for stress-related disorders such as PTSD in daily clinical practice. 

The social defeat stress (SDS) paradigm is widely used in rodents as a highly validated animal model of bullying. By using the resident–intruder model, which includes social subordination, threat, and physical abuse, it is possible to reproduce and study the main features of bullying in humans [28,29]. Generally, this paradigm consists of placing an experimental rodent (intruder) into the territory of a larger and aggressive conspecific (resident) that attacks the intruder for one or more sessions [30]. Compelling evidence indicates that SDS can have both short- and long-term consequences on emotional and cognitive domains, such as increased anxiety and altered cognitive flexibility, in adult rodents. Exposure to adverse stimuli, particularly during sensitive windows of brain development, is a critically important environmental factor involved in an individual’s response to stress [31]. Individuals can be classified into two categories on the basis of their reaction to stress: vulnerable people that negatively respond to adverse stressful stimuli, and resilient people that positively respond to the same stimuli [32,33]. Various hypotheses have been postulated to explain this difference between individuals. According to the cumulative stress theory, individuals that are continuously exposed to stress across their life span are more predisposed to develop psychopathologies [34]. Conversely, in the match/mismatch hypothesis, vulnerability or resilience to stress depends on the interaction between early-life and adult stress. The two-hit stress model has been used to investigate whether exposure to two different stressors at different ages increases or decreases the risk of developing psychopathologies after experiencing the second stressor [35,36,37,38,39,40]. The effects of social stress similar to bullying in humans experienced during adolescence on the reaction to an additional stressor later in life, however, are less investigated [41].

To elucidate this issue, we exposed rats to SDS during early adolescence and then exposed them in adulthood to a single prolonged stress (SPS) as a second stressor. SPS is a validated stress paradigm comprising three stressful events presented during a single session [42,43,44]. We evaluated whether exposure to stress during early adolescence (first hit) affects emotionality and cognitive processes in the long-term, and whether exposure to a second challenge (second hit) later in life alters such effects. Rats were exposed to SDS during early adolescence and to SPS in adulthood, and then subjected to a behavioral test battery, including the open field, acoustic startle response, Morris water maze, and auditory fear conditioning. Further, to study the interplay between stress and BDNF in these effects, we examined hippocampal BDNF protein expression and plasma corticosterone levels in adulthood. Further, we evaluated the long-term effects induced by the two stressors on BDNF levels in the brain region most critically affected by stress effects, i.e., the hippocampus. We then evaluated whether any hippocampal BDNF alterations were paralleled by changes in plasma corticosterone concentrations.

## 2. Materials and Methods

### 2.1. Animal Care 

Sprague Dawley rats (Charles River Laboratories, Calco, Italy) were housed in air-conditioned vivarium rooms (temperature: 21 ± 1 °C; lights on from 7:00 am to 7:00 pm) with pellet food and water available ad libitum. Adolescent male rats were housed in groups of four per cage until postnatal day (PND) 70 when animals of the same treatment were pair-housed. Adult Sprague Dawley male rats 400–450 g (Charles River Laboratories, Calco, Italy), used as residents for the SDS protocol, were housed with a sterile, but sexually receptive adult Sprague Dawley female rat to enhance their aggressiveness towards the intruders [45]. All the experiments were performed during the light phase of the cycle (10:00 AM–3:00 PM) and were in compliance with the ARRIVE guidelines, the Directive 2010/63/EU of the European Parliament, the D. L. 26/2014 of Italian Ministry of Health, and approved by the Italian Ministry of Health.

### 2.2. Social Defeat Stress

We used a slightly modified SDS protocol previously described [28,46]. Briefly, for seven consecutive days (PND 28–34), adolescent intruders were placed in the resident’s home cage (the female rat was removed for the duration of the encounter). Each session began with a period of investigation and free interaction until 15 min had elapsed. Rats were then separated by a wire mesh barrier (1-cm weave), allowing continued olfactory and visual contact, but preventing physical contact for the duration of the 30-min session. Controls were picked up from the resident’s home cage and then returned to their home cage. The resident–intruder paradigm was repeated daily for 7 days with the intruders being exposed to a different resident each day to prevent any habituation to the resident aggressor. Residents that failed to consistently attack or that were overly aggressive and caused injury to intruders were not used. 

### 2.3. Single Prolonged Stress Protocol

We used a previously described SPS protocol [43,47,48] with slight modification. Briefly, rats at PND 90 were exposed to a single session of SPS that consisted of 2 h of restraint stress in a restrainer (30 cm in length with an inside diameter of 7.5 cm), 15 min of forced swim stress (24 ± 1 °C water temperature) followed by 15 min of recovery, and then isoflurane exposure until loss of consciousness. Rats not assigned to SPS groups were placed in a separate room throughout the duration of SPS. After the SPS procedure, all rats were returned to their home cage and left undisturbed for 30 days.

### 2.4. Experimental Design

**Figure 1:**
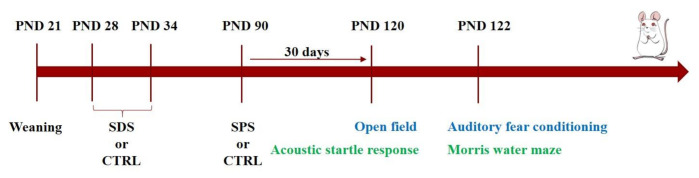
Experimental timeline. Separate cohorts of male rats at PND 28 were randomly assigned to either the CTRL-CTRL, SDS-CTRL, CTRL-SPS, or SDS-SPS groups. Rats were subjected to 30 min social defeat stress (SDS) daily for seven consecutive days from postnatal day (PND) 28 to 34, and then to the single prolonged stress (SPS) paradigm in adulthood (PND 90). Thirty days after SPS exposure, two separate groups of rats were exposed to a behavioral test battery. The first group was exposed to open field (PND 120) and auditory fear conditioning (PND 122) tasks, and the second one was exposed to acoustic startle response (PND 120) and Morris water maze (PND 122) tasks. One week after the last behavioral task, rats were killed, and the bilateral hippocampus was immediately dissected from each animal for biochemical analyses.

As shown in Figure 1, adolescent rats at PND 28 were randomly assigned to one of four groups:

- CTRL-CTRL group: rats not exposed to any stressors
- SDS-CTRL group: rats exposed to only SDS during the adolescence (PND 28-34)
- CTRL-SPS group: rats exposed to only SPS at PND 90
- SDS-SPS group: rats exposed to SDS during adolescence and SPS at PND 90

Animals were left undisturbed for 30 days after SPS exposure and divided into two different cohorts and then exposed to two behavioral test batteries (see Figure 1). 

### 2.5. Behavioral Tests

#### 2.5.1. Open Field 

In the open field test, each rat was placed into the corner of the open field arena (80 × 80 × 60 cm^3^). The test was performed under dim light conditions (2 lux) and lasted 20 min, as previously described [49]. The total distance traveled (cm) by each rat and the time spent in the center of the arena were determined as parameters indicating the rat locomotor activity and emotional reactivity, respectively. After each session, rats were returned to their home cage, fecal boli were removed, and the walls and floor of the arena were cleaned with a 70% ethanol solution. The total distance traveled was acquired and analyzed using an automated video-tracking system (Smart, Panlab, Barcelona, Spain).

#### 2.5.2. Acoustic Startle Response 

The acoustic startle response was measured as previously described [47] with slight modification. Rats were placed in a startle reflex apparatus (Med Associates, Fairfax, VT, USA) for a 5-min acclimatization period with a 70-dB background noise, which continued for the entire session. Each session consisted of 22 pulse trials (115 db) with intertrial intervals selected randomly between 10 and 15 s. Acoustic devices and startle cages were connected to a computer, which detected and analyzed all chamber variables using customized software (Med Associates). The system allows recording and analysis of the signal generated by the animal movement through a high-sensitivity weight transducer system. The mean startle reflex response for each animal was calculated as the average of the responses to the 22 pulse trials. 

#### 2.5.3. Morris Water Maze

The experimental apparatus was a circular tank (1.83 m in diameter and 0.58 m in height) filled with water (23–24 °C) to a depth of 20 cm. The maze was located in a room containing many salient, visual, extra-maze cues. During the spatial training a rectangular platform (20 × 25 cm^2^) was placed at a fixed location 25 cm away from the edge of the pool and 2.5 cm below the water surface. The experiments were performed according to a previously described procedure [50,51]. For spatial training, the rats were given four trials on each daily session for three consecutive days. On each trial, the animal was placed in the tank facing the wall at one of the four designated start positions and allowed to escape onto the hidden platform. If an animal failed to find the platform within 60 s during the first day of the training, it was manually guided to the platform. The rat was allowed to remain on the platform for 10 s and was then placed into a holding cage for 25 s until the start of the next trial. The time each animal spent to reach the platform was recorded as the escape latency. For the acquisition phase, the average of escape latencies across the trials during each day of training was analyzed. Retention of the spatial training was assessed 24 h after the last training session with a 60-s free-swim probe trial and a new starting position was used. Training and probe trials were videotaped, and an automated tracking system (Smart, Panlab, Barcelona, Spain) was used to analyze the swim path of each subject and calculate the initial latency to cross the platform location and the number of crossings through the platform location. The target and opposite quadrants were equidistant from the starting position on the probe trial. 

#### 2.5.4. Auditory Fear Conditioning

We used a slightly modified protocol as previously described [52,53]. All phases of the auditory fear conditioning task were performed in the same but contextually modified operant chambers, located in sound-attenuating cubicles (Med Associates, VT, USA). The floor of the chambers consisted of stainless-steel bars that delivered a scrambled electric footshock. On day 1, rats were exposed to chamber A and received five habituation tones (30 s, 4 kHz, 75 dB; 3 min intertrial interval), immediately followed by seven conditioning tones that co-terminated with footshocks (0.5 s, 0.65 mA). On day 2, rats were exposed to chamber B (contextually modified with black walls) for the extinction session, which consisted of 12 tones (30 s, 4 kHz, 75 dB; 3 min intertrial interval) in the absence of footshock. On day 3, rats were returned to the chamber B and presented with eight tones (30 s, 4 kHz, 75 dB; 3 min intertrial interval) in the absence of footshock to test for extinction retrieval. Freezing behavior, defined as the absence of all movements except for those related to breathing [54], served as the measurement of fear and was monitored with digital video cameras. An experimenter blinded to the experimental conditions quantified the total time that rats spent freezing during the 30-s tone presentations by using a digital stopwatch. The percentage of time spent in freezing during the 30-s tone presentations was calculated as: [time spent in freezing during the tone presentation (s)/30 s] × 100. For the conditioning session (day 1), the time spent in freezing during the first and the last tone presentation was represented. For the extinction session (day 2) the average of time spent in freezing during the three blocks of four trials each was evaluated, while for the retrieval session (day 3) the average of percentage of time spent in freezing during all trials was represented.

### 2.6. Biochemical Analyses

#### 2.6.1. Tissue Collection and Western Blotting Analysis

Western blotting analysis was performed as previously described [55,56]. Rats were decapitated after seven days from the last behavioral task to measure BDNF expression at baseline levels. The hippocampus was rapidly collected, pooled from both sides of the brain, and homogenized in lysis buffer, containing Tris-HCl (pH 8) 50 mM, NaCl 150 mM, NP40 1%, SDS 0.1%, and a protease inhibitor mixture (Sigma, St. Louis, MO, USA, P8340, 1/100) in distilled water. Tissues were centrifuged at 15,000 rpm for 10 min, and the supernatant removed and stored in aliquots at −80 °C until use. Equivalent amounts (30 μg) of each sample calculated by Bradford assay were resolved on 12% acrylamide SDS-PAGE gels, and then transferred onto nitrocellulose. Membranes were blocked for 1 h at room temperature in a solution containing 5% non-fat dry milk (Bio Basic, Markham, ON, Canada) in tris-buffered saline (TBS) 0.1% tween 20 (TBS-T). Membranes were then incubated overnight at 4 °C with anti BDNF rabbit polyclonal antibody (1:500, Bioss Antibodies, Boston, MA, USA) or anti β-actin goat polyclonal antibody (1:500, Biorbyt Ltd., Cambridge, UK). After being washed three times for 10 min in TBS-T, membranes were incubated for 1 h at room temperature in the proper secondary horseradish peroxidase-conjugated antibodies (HRP-conjugated goat anti-rabbit IgG, 1:10,000, Thermo Fisher Scientific, Rockford, IL, USA; HRP-conjugated mouse anti-goat IgG, 1:10,000, Santa Cruz Biotechnology, Santa Cruz, CA, USA). Immunoreactivity was developed with enhanced chemiluminescence (ECL system; BioRad, CA, USA). For analysis of the Western blotting data, densitometric analysis was performed using Image J software. 

#### 2.6.2. Plasma Corticosterone Levels

Rats were decapitated after seven days from the last behavioral task in order to measure baseline corticosterone levels. Trunk blood was collected after decapitation (11:30 AM–13:30 PM) in tubes containing 200 μL of 0.1M EDTA and samples were centrifuged at 1000× *g* for 15 min at 4 °C. Plasma was stored at −20 °C and analyzed for corticosterone levels using a DetectX ELISA kit (Arbor Assays, Ann Arbor, MI, USA) according to the manufacturer’s instructions, as previously described [57,58]. 

### 2.7. Statistical Analysis 

Statistical analysis was performed using SPSS statistical software. Each measure is expressed as mean ± SEM. A repeated measures ANOVA (RM ANOVA) was used to evaluate the mean escape latency across trials during each training day in the Morris water maze and the percentage of freezing during the conditioning and extinction phases of the auditory fear conditioning task. In all other cases, data were analyzed with two-way ANOVA when appropriate. Biochemical data were analyzed with a two-way ANOVA. The Tukey–Kramer post-hoc test was performed to control for significant differences between groups when appropriate. Significance was considered for *p* < 0.05.

## 3. Results

### 3.1. The Interaction Between Social Defeat Stress (SDS) during Early Adolescence and Single Prolonged Stress (SPS) in Adulthood Induces Resilience towards the Development of Hyperarousal and Anxiety Later in Life

#### 3.1.1. Open Field Test

**Figure 2:**
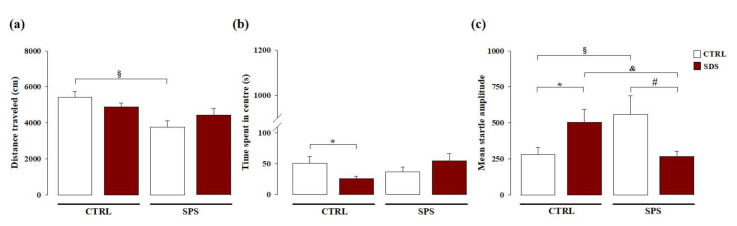
Long-term effects on locomotor activity, hyperarousal, and anxiety-like behavior induced by SDS, SPS, or the combination of both stressors. Long-term effects induced by SDS, SPS, or both stressors on the (**a**) distance traveled (cm) evaluated in the open field arena, (**b**) time spent in the center of the arena in the open field, and (**c**) mean startle amplitude in the acoustic startle response task. *, *p* < 0.05 vs. CTRL-CTRL group; §, *p* < 0.05 vs. CTRL-CTRL group; &, *p* < 0.05 vs. SDS-CTRL group; #, *p* < 0.05 vs. CTRL-SPS group. (N = 8–12 rats per group or N = 8–11 rats per group, in the open field or acoustic startle response tasks, respectively).

We evaluated whether the exposure to SDS during early adolescence and/or to SPS in adulthood induced enduring alterations on locomotor activity and emotionality. As shown in Figure 2a, two-way ANOVA for the distance traveled in the open field showed no significant effect of SDS (F_(1,35)_ = 0.205; *p* = 0.653), a significant effect of SPS (F_(1,35)_ = 5.291; *p* = 0.028), and no significant SDS × SPS interaction (F_(1,35)_ = 2.726; *p* = 0.108). Post hoc analysis revealed that rats exposed to only SPS traveled shorter distance compared with control rats (*p* < 0.05). Two-way ANOVA showed no significant main effects for the time spent in the center of the open field arena for SDS (F_(1,35)_ = 0.141; *p* = 0.710) and SPS (F_(1,35)_ = 0.551; *p* = 0.463), but revealed a significant SDS × SPS interaction (F_(1,35)_ = 4.631; *p* = 0.038, Figure 2b). Post hoc analysis revealed that rats exposed to only SDS spent less time in the center of the open field arena compared with control rats (*p* < 0.05). These results indicate that SDS experienced during early adolescence does not affect locomotor activity in adulthood, but does induce an anxious-like profile in adult rats. Conversely, rats exposed to SPS in adulthood and tested in the open field test exhibited reduced locomotor activity, but no alterations of emotional parameters. Interestingly, rats subjected to both stressors exhibited no alterations in locomotor activity or emotionality, indicating that SDS in adolescence combined with SPS experienced later in life confers resilience to stress.

#### 3.1.2. Acoustic Startle Response

We evaluated whether SDS during early adolescence, SPS in adulthood, or the combination of both stressors may induce long-term effects on hyperarousal and anxiety-like behavior. As shown in Figure 2c, two-way ANOVA for the mean startle amplitude showed no significant effect of SDS (F_(1,33)_ = 0.170; *p* = 0.683), or SPS (F_(1,33)_ = 0.064; *p* = 0.801), but a significant effect of SDS × SPS interaction (F_(1,33)_ = 11.166; *p* = 0.002). Post hoc analysis revealed that rats singularly exposed to SDS or to SPS had a higher mean startle amplitude than control rats (*p* < 0.05). Conversely, rats subjected to both stressors showed lower mean startle amplitude than rats only exposed to SDS or to SPS (*p* < 0.05) and similar to that of control rats never exposed to any stressor. These results indicate that rats singularly exposed to SDS during early adolescence or to SPS in adulthood and tested in the startle apparatus show hyperarousal and increased anxiety later in life and that such effects are absent when rats are subjected to both stressors, thus demonstrating again that SDS during early adolescence together with the second challenge experienced later in life promotes resilience towards emotional alterations. 

### 3.2. The Interaction between SDS during Early Adolescence and SPS in Adulthood Induces Resilience towards Spatial Memory Deficits Later in Life 

**Figure 3:**
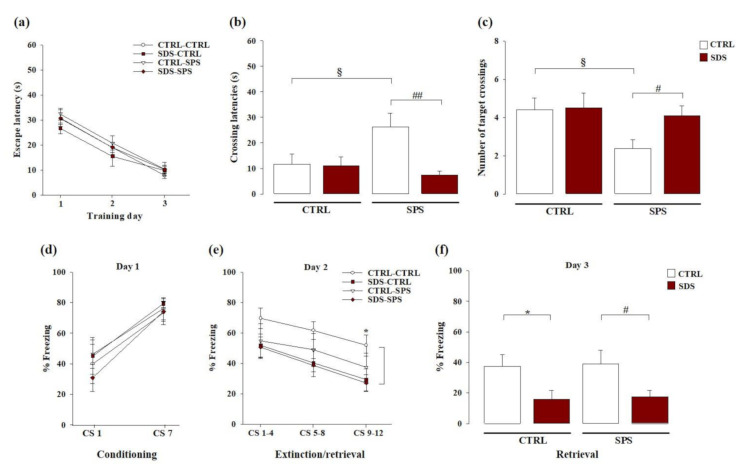
Long-term effects on spatial and cued fear memory dynamics induced by SDS, SPS, or the combination of both stressors. Long-term effects induced by SDS, SPS, or both stressors in the Morris water maze on (**a**) escape latencies across trials during each day of the acquisition phase, (**b**) the latency to cross the platform location, and (**c**) the number of crossings over the platform location during the probe session. Long-term effects induced by SDS, SPS, or both stressors in the auditory fear conditioning paradigm on (**d**) percentage of time spent in freezing during the first and last tone presentations at conditioning (day 1), (**e**) mean percentage of time spent in freezing during the three blocks of four trials each at extinction (day 2), (**f**) mean percentage of time in spent freezing during all eight trials at extinction retrieval (day 3). *, *p* < 0.05 vs. CTRL-CTRL group; §, *p* < 0.05 vs. CTRL-CTRL group; #, *p* < 0.05 vs. CTRL-SPS group; ##, *p* < 0.01 vs. CTRL-SPS group. (N = 8–11 rats per group or N = 8–12 rats per group, in the Morris water maze or auditory fear conditioning tasks, respectively).

We examined whether SDS during early adolescence and/or SPS in adulthood may alter spatial memory in the long-term and whether these effects are altered when rats experienced both stressors in the Morris water maze task. As shown in Figure 3a, RM ANOVA for escape latency during spatial training revealed a significant effect of trials (F_(2,66)_ = 86.483; *p* < 0.0001), indicating that all groups progressively learned to locate the platform across the three training days (no other statistical differences were found). 

For retention memory, during the probe trial two-way ANOVA for the initial latency to cross the platform location indicated a significant main effect of SDS (F_(1,33)_ = 7.065; *p* = 0.012), no significant effect of SPS (F_(1,33)_ = 2.248; *p* = 0.143), but a significant interaction between these two factors (F_(1,33)_ = 6.279; *p* = 0.017; Figure 3b). Post hoc analysis revealed that the latency to cross the platform location of rats exposed to only SPS was higher than that of the control group (*p* < 0.05) and those of rats subjected to both stressors (*p* < 0.01). Two-way ANOVA for the number of crossings through the platform location revealed no significant effect of SDS (F_(1,33)_ = 2.260; *p* = 0.142), a significant main effect of SPS (F_(1,33)_ = 4.061; *p* = 0.052), and a tendency towards significance for the SDS × SPS interaction (F_(1,33)_ = 1.790; *p* = 0.190; Figure 3c). Post hoc analysis indicated that the crossings through the platform location of rats only exposed to SPS were lower than those of controls (*p* < 0.05) and those of rats subjected to both stressors (*p* < 0.05). These results demonstrate that rats singularly exposed to SPS had spatial memory retention deficits. Rats that experienced both SDS during early adolescence and SPS in adulthood did not show these cognitive deficits. 

### 3.3. The Interaction Between SDS during Early Adolescence and SPS in Adulthood Induces Vulnerability towards Cued Fear Memory Deficits Later in Life 

We evaluated whether SDS during early adolescence and/or SPS in adulthood alter cued fear memory dynamics in adulthood and whether the exposure to both stressors may affect these effects in the auditory fear conditioning task. As shown in Figure 3d, RM ANOVA for the percentage of freezing during fear conditioning acquisition (day 1) revealed a significant main effect of tone presentation (F_(1,35)_ = 42.958; *p* < 0.0001), indicating that all groups learned to associate the tone with the footshock (no other statistical differences were found). RM ANOVA for the time spent in freezing during the memory extinction phase on day 2 (Figure 3e) revealed significant main effects of tone presentation (F_(1,35)_ = 14.625; *p* < 0.0001) and SDS (F_(1,35)_ = 4.692; *p* = 0.037), but no significant main effect of SPS (F_(1,35)_ = 1.389; *p* = 0.247) or SDS × SPS interaction (F_(1,35)_ = 0.885; *p* = 0.353). No other statistical differences were found. Post hoc test revealed that rats only exposed to SDS froze less than controls during the last four tones presentation (*p* < 0.05). Two-way ANOVA for the time spent in freezing during the memory retrieval session on day 3 (Figure 3f), showed a significant main effect of SDS (F_(1,35)_ = 10.089; *p* = 0.003), but no significant main effect of SPS (F_(1,35)_ = 0.055; P = 0.817) or SDS × SPS interaction (F_(1,35)_ = 0.0001; *p* = 0.991). Post hoc analysis showed that rats only exposed to SDS and rats exposed to both stressors presented less mean percentage of freezing as compared with control rats and rats exposed to only SPS, respectively (*p* < 0.05). These results indicate that exposure to SDS during early adolescence altered extinction and extinction retrieval in adult rats, while SPS did not. Further, when rats were exposed to both stressors fear memory dysfunctions induced by SDS persist, thus indicating that SDS combined with SPS produces vulnerability towards fear memory dynamics. 

### 3.4. The Interaction Between SDS during Early Adolescence and SPS in Adulthood Normalizes Protein Levels of Brain-Derived Neurotrophic Factor (BDNF) Within the Hippocampus Later in Life 

**Figure 4:**
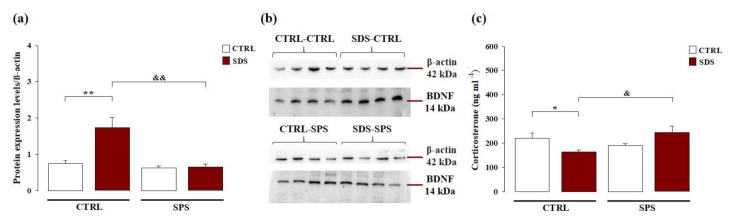
Long-term effects on hippocampal brain-derived neurotrophic factor (BDNF) protein expression and plasma corticosterone levels in rats exposed to SDS, SPS, or the combination of both stressors. (**a**) Western blotting analysis of hippocampal BDNF protein expression levels in adult rats. Data represent means (±SEM) from three replicate experiments and are normalized with β-actin and (**b**) representative Western blot bands of four samples. (**c**) Plasma corticosterone levels. Data represent means (±SEM) from three replicate experiments. *, *p* < 0.05 vs. CTRL-CTRL group; **, *p* < 0.01 vs. CTRL-CTRL group; &, *p* < 0.01 vs. SDS-CTRL group; &&, *p* < 0.01 vs. SDS-CTRL group. (N = 6–8 per group).

We examined whether exposure to SDS during early adolescence and/or SPS in adulthood modulates BDNF protein expression levels within the hippocampus in adulthood. We performed Western blot analysis to compare BDNF protein expression levels within the hippocampus of all four groups of tested rats. As shown in Figure 4a, two-way ANOVA for hippocampal BDNF levels showed significant effects of SDS (F_(1,28)_ = 11.074; *p* = 0.003), SPS (F_(1,28)_ = 15.960; *p* < 0.001), and interaction between these two factors (F_(1,28)_ = 10.124; *p* = 0.004). Post hoc analysis revealed that rats exposed to only SDS have increased hippocampal BDNF protein expression compared with control rats (*p* < 0.01) and rats exposed to both stressors (*p* < 0.01). These results indicate that exposure to SDS during early adolescence induced an increase in BDNF protein expression in the hippocampus of adult rats, while SPS did not. Moreover, these alterations induced by SDS were absent in rats exposed to both stressors. The representative Western blot bands are shown in Figure 4b. 

### 3.5. The Interaction between SDS during Early Adolescence and SPS in Adulthood Normalizes the Corticosterone Plasma Levels Later in Life 

Long-term effects of the exposure to SDS during early adolescence and/or SPS in adulthood on corticosterone plasma levels were evaluated. As shown in Figure 4c, two-way ANOVA for plasma corticosterone levels did not reveal a significant main effect of SDS (F_(1,20)_ < 0.001; *p* = 0.988) or SPS (F_(1,20)_ = 2.017; *p* = 0.171), but a significant interaction between these two factors (F_(1,20)_ = 9.889; *p* = 0.005). Post hoc analysis revealed that rats exposed to only SDS had lower corticosterone plasma levels than control rats (*p* < 0.05) and rats subjected to both stressors (*p* < 0.05). These results indicate that exposure to SDS during early adolescence, but not to SPS in adulthood or to both stressors, induced low plasma corticosterone levels in adult rats. 

## 4. Discussion

The present findings indicate that exposure to social stress during early adolescence influences the ability to cope with a second challenge experienced later in life. Specifically, rats exposed to SDS during early adolescence and then to SPS in adulthood exhibited resilience against the development of alterations in emotionality, arousal, and spatial memory, but demonstrated vulnerability toward cued fear memory dysfunction. In addition, rats exposed to both stressors showed resilience toward alterations induced by SDS exposure on the hippocampal BDNF protein expression and plasma corticosterone levels. These effects seemed to be bidirectional; SPS alone induced alterations of locomotor activity and spatial memory retention that were not detected in rats that experienced SDS during adolescence. 

SDS exposure affects both the emotional and cognitive domains in rodents, and these effects are profound and enduring when the social stress occurs during important developmental stages [59,60]. SDS is a validated experimental model that mimics some of the effects of bullying victimization in humans [28,29], a stressor that frequently occurs among school-age adolescents. Adolescent victims of bullying are characterized by an enhanced risk for developing mental disorders later in life, such as anxiety, depression, and PTSD [27,61]. Not all adolescents experiencing stressful situations, however, develop psychiatric diseases in adulthood [62,63]. According to the match/mismatch hypothesis, vulnerability or resilience to developing a psychopathology in adulthood depends by how early-life stressful events match the adversities in later life [3,4,5,6]. Here, by using the resident–intruder paradigm (SDS) as a first stressor and an acute traumatic stress (SPS) as the second stressor, we assessed whether the interaction between SDS during early adolescence and SPS in adulthood alters the vulnerability or resilience towards long-term emotional and cognitive function. In accordance with previous studies [59,60], we found that adolescent rats exposed to chronic social defeat for seven days developed long-term emotional impairments in adulthood, as indicated by a reduced time spent in the center of the open field arena and an enhanced startle response. Moreover, SDS during adolescence was linked to distinct patterns of cognitive impairment. We found impairments in cued fear memory, while spatial memory was not affected. Cued fear conditioning is widely used to measure emotional learning and memory [64,65,66]. In this behavioral paradigm, animals learn to relate an aversive event, such as a footshock, with a stimulus, such as a tone, and subsequently express fear responses after stimulus presentations. Consistent with our results indicating that SDS during early adolescence altered cued fear memory alterations in the long-term, Novick and colleagues reported decreased freezing in rats exposed to SDS during adolescence and tested in the fear conditioning task in adulthood, indicating impaired fear learning [67]. In particular, our results demonstrated that fear memory alterations are referred to the extinction phase and not to the consolidation one, as indicated by the lower freezing percentage during the last tones presentation exhibited by adult rats exposed to SDS in adolescence with respect to controls. Interestingly, we found that adult rats subjected to SDS during early adolescence did not differ from controls in locomotor activity in a novel environment. Consistent with our finding, previous evidence demonstrated that subjecting juvenile rats to SDS does not alter locomotor activity in the open field in adulthood [68]. In contrast to previous studies demonstrating that SDS in adult rats impairs spatial memory function [69,70,71], we found that exposure to SDS during early adolescence did not produce long-term effects on spatial memory, suggesting that some of the effects of SDS exposure may be an acute response to stress and are not long-lasting.

The SPS paradigm is a well-validated rodent model used to reproduce some of the hallmark symptoms observed in PTSD patients (e.g., hyperarousal, anxiety, and spatial and fear memory deficits) [72,73,74,75]. The manifestation of a PTSD-like phenotype following SPS exposure is time-dependent and requires 7–14 days of incubation to develop [44,76,77]. Because PTSD is a chronic debilitating psychiatric disease, the majority of preclinical studies that use SPS as a rodent model of PTSD do not evaluate whether behavioral alterations persist long after trauma [72,73,74,75]. In the present work, rats were exposed to SPS at PND 90 and the behavioral outcomes were evaluated at 1 month after trauma exposure. As we previously demonstrated, SPS exposure induced reduced locomotor activity, hyperarousal, anxiety-like behavior, and spatial memory retention deficits long after exposure to a stressful condition [48]. This is consistent with findings from a previous study demonstrating a sustained reduction in exploratory behavior after exposure to stress [49] and with clinical data indicating an exaggerated startle response as a characteristic trait of PTSD patients [78,79]. Hyperarousal is a state of excessive vigilance towards different stimuli, and although the real danger may not be present, PTSD patients act as if it is, thus causing distress and impaired social abilities [80]. It is important to note that these SPS-induced effects did not persist when rats were previously exposed to SDS during early adolescence, thus indicating a positive interaction between the two stressors with the social stress experienced during adolescence altering the ability of the rats to cope with additional trauma in adulthood, and thereby leading to increased resilience against stress-induced alterations.

Studies using animal models of early-life stress demonstrate that an imbalance between stress mediators leads to emotional and cognitive impairments [81]. Adolescence is a period of impressive brain maturation in which the structure of the brain is ever-changing [8]. Thus, maintaining a correct balance between mediators that sustain synaptic plasticity is critical. In the present work, we found that SDS experienced during early adolescence enhances protein expression of hippocampal BDNF and reduces corticosterone plasma levels with respect to control animals, and that these effects are abolished by a combination of SDS and SPS. Exposure to SDS or chronic mild stress increases BDNF expression in young rats, but not in adult rats, who exhibit decreased hippocampal BDNF expression [82,83]. No data are available on the enduring effects induced by SDS on BDNF expression and, to the best of our knowledge, this is the first evidence demonstrating that adult rats exposed to SDS during early adolescence, but not in adulthood, exhibit increased expression of BDNF in the hippocampus that is linked to behavioral alterations (e.g., hyperarousal, anxiety, and cued fear memory deficits). Similar to our results, previous studies showed increased hippocampal expression of polysialylated neuronal cell adhesion molecule, an important key plasticity molecule [84,85] in adult rats previously exposed to peripubertal [86] or juvenile stress [87], indicating that exposure to early-life stress may disrupt hippocampal maturation. BDNF is one of the most important neurotrophic factors thus playing an important role in the modulation of neuronal morphology and synaptic plasticity, particularly within the hippocampus [88]. Previous evidence indicated that hippocampal BDNF expression reaches its highest level during specific critical windows for brain development, such as early-adolescence [89,90]. Of note, BDNF is strictly involved in the regulation of other neurobiological mediators (e.g., endocannabinoids, glucocorticoids) [91,92] which in turn modulate several brain functions. For all these reasons and on the basis of the present finding we hypothesize that altered hippocampal BDNF expression may represent one possible mechanism responsible for the long-term behavioral alterations induced by SDS, suggesting that exposure to social stress during early adolescence may affect hippocampal maturation processes, leading to increased hippocampal BDNF levels later in life. Glucocorticoids play an important role in the coping mechanisms in response to stress and modulate BDNF expression in stress-sensitive brain areas such as the hippocampus [18,20]. Moreover, glucocorticoids and BDNF modulate synaptic plasticity mechanisms by inducing opposing effects [17,20]. Here we found that the increased BDNF levels in adult rats exposed to SDS in early adolescence are paralleled by lower corticosterone plasma levels compared with non-stressed animals or rats exposed to both stressors. 

Our results have strong translational value because clinical studies demonstrate that patients with PTSD [93] or depression [94] who experienced childhood trauma have low baseline levels of cortisol. In conclusion, our data indicate that exposure to SDS during early adolescence or to SPS in adulthood experience different effects later in life. In adult rats exposed to both SDS and SPS, the later development of emotional and cognitive alterations strongly depends on the interaction between the early-life challenge and a second challenge later in life. Our study provides important information about a possible mechanism involved in stress-resilience and susceptibility as a consequence of a combination of two stressors at different periods of life. Understanding the neurobiological underpinnings of stress-induced alterations in animal models is extremely relevant in terms of translational value since it may open the way to the identification of new potential pharmacological interventions to treat stress-related disorders and notably to prevent the development of these diseases after experiencing a second trauma later in life. To the best of our knowledge, this study is the first to evaluate whether exposure to an early-life stress protocol that mimics bullying in adolescents affects the development of psychopathologies in adulthood in response to a trauma experienced later in life. 

## Data Availability

Not applicable.
